# Association of Gene Polymorphisms with Breast Cancer Risk in the Kazakh Population

**DOI:** 10.31557/APJCP.2023.24.12.4195

**Published:** 2023

**Authors:** Nasrulla Shanazarov, Yerbol Zhapparov, Raushan Kumisbekova, Dinara Turzhanova, Nargiz Zulkhash

**Affiliations:** 1 *Department of Strategic Development, Science and Education, Medical Centre Hospital of President’s Affairs Administration of the Republic of Kazakhstan, Astana, Republic of Kazakhstan. *; 2 *Center for Photodynamic Therapy, Medical Centre Hospital of President’s Affairs Administration of the Republic of Kazakhstan, Astana, Republic of Kazakhstan. *; 3 *Clinical and Diagnostic Department, “UMIT” International Oncological Tomotherapy Center, Astana, Republic of Kazakhstan. *; 4 *Department of Chemotherapy, Multidisciplinary Medical Center of the Akimat of Astana, Astana, Republic of Kazakhstan. *; 5 *Department of Radiology named after Academician Zh.Kh. Khamzabaev, Astana Medical University, Astana, Republic of Kazakhstan. *; 6 *Department of Public Health, Astana Medical University, Astana Medical University, Astana, Republic of Kazakhstan. *

**Keywords:** Single, nucleotide polymorphisms, cancer marker, breast adenocarcinoma, genotype, gene mutations

## Abstract

**Objective:**

The research aim is analyzing and identify reliable genetic markers of breast cancer risk in the Kazakh population.

**Methods:**

The databases were analyzed with the selection of polymorphisms associated with the development of breast cancer and further genotypic study of a group of women with a confirmed diagnosis of breast adenocarcinoma (group No. 1) and a group of relatively healthy women (group No. 2).

**Result:**

The research presents the results of a study on the frequency of certain single-nucleotide polymorphisms in patients with breast cancer in the Republic of Kazakhstan. The frequency of single-nucleotide polymorphisms rs4646, rs1065852, rs4244285, rs67376798, rs6504950, rs2229774, rs1800056, rs16942, rs4987047 is statistically significant compared to the control group of patients. These polymorphisms in the Kazakh population have a direct association with an increased risk of breast cancer in women and may be used as cancer indicators during the genetic screening of patients with a complicated family history. Single-nucleotide polymorphisms such as rs55886062, rs3918290, rs12721655, rs4987117, rs2229774, rs11203289, rs137852576, rs11571833, rs80359062 and rs11571746 were found in more than 40. Zero percent of patients with breast cancer may be used as markers for detecting patients at increased risk of breast malignancy in the Kazakh population without a history of poor family history.

**Conclusion:**

The usage of the data obtained in a set of state programs for early screening of patients will improve the rates of early breast tumor detection, form groups of patients with a high risk of disease development and improve the quality and expectancy of life.

## Introduction

Currently, breast cancer (BC) is the most common cancer diagnosed in women of reproductive age in developed countries (Ferlay et al., 2019), which is often associated with high mortality rates (Fahad Ullah, 2019; Momenimovahed and Salehiniya, 2019). Under the International Agency for Research on Cancer 2020, more than two million cancer cases of various etiologies are diagnosed worldwide each year (Ahmad, 2019), among which more than 35% have lethal outcomes (Lei et al., 2021). Long-term research on mortality indexes among breast cancer patients in 195 countries show the tendency for a gradual increase in the mortality process over the last 25 years (Azamjah et al., 2019). Every fifth tumor diagnosed in women of the Republic of Kazakhstan is a breast tumor (Igissinov et al., 2019). Every year in Kazakhstan about five thousand new cases of breast cancer are registered (Toguzbayeva et al., 2021), which is connected both with the increase in population, i.e., normal demographic processes, and with effective state programs of mammalogical screening for early diagnostics of breast cancer. Despite disease heterogeneity (Ferlay et al., 2019; Januškevičienė and Petrikaitė, 2019), the etiology of breast cancer is related to several genetic, epigenetic, and phenotype factors (Coughlin, 2019). Epigenetic factors, encompassing DNA methylation, histone modifications, and non-coding RNA regulation, significantly influence breast cancer development by altering gene expression. These epigenetic changes can act in conjunction with genetic risk factors and are modulated by environmental influences. Recognizing the interplay between genetic and epigenetic factors is crucial for a comprehensive understanding of breast cancer risk and may inform more personalized prevention and treatment strategies.

At the same time, in addition to genetic factors, environmental factors that increase the risk of developing BC play an important role (Louro et al., 2019). These include a long history of hormone therapy, regular alcohol consumption, obesity, and physical inactivity (Ginsburg et al., 2020). There are also ethnic peculiarities of BC occurrences (Britt et al., 2020), for example, the average age of pathology development; for the Kazakh population, it is on average 50 years (Toguzbayeva et al., 2021), with the peak of diagnostics and treatment of pathology at 65-69 years. The research of genetic polymorphisms is crucial in comprehending the pathogenesis and course of BC for each patient individually (Pashayan et al., 2020). The accurate genetic and molecular diagnosis with the immunophenotypic profile of the tumor (Moo et al., 2018) allows the use of modern personalized treatment protocols for patients with BC.

Genotypic research of single nucleotide polymorphisms (SNPs) in patients with cancer allows the formation of high-risk groups with more aggressive disease, and during population screening - to identify patients with probable risks of developing specific types of cancer neoplasms (Thorat and Balasubramanian, 2020). Detection of specific gene mutations also allows the application of preventive measures to prevent cancer processes of both invasive and conservative types with regular monitoring of the patient’s condition for active monitoring. Individual screening allows the reduction of morbidity rates by more than 15%, significantly improving the index of quality of life and health (Tang et al., 2018). SNP genotyping is performed with high accuracy for patients of all ages and somatic conditions and is the method of choice for predicting the risks of developing specific types of cancer. Results of the Breast Cancer Research Foundation in the United States research show that testing and early screening of BC significantly improve clinical management strategies for women in the general population with inherited pathogenic variants in specific genes peculiar to the population. For example, according to published data (Hu et al., 2021), pathogenic variants in BARD1, RAD51C, and RAD51D were statistically likely associated with an increased risk of estrogen-receptor-negative and triple-negative forms of breast cancer, whereas pathogenic variants in ATM, CDH1, and *CHEK2* were associated with an increased risk of estrogen-receptor-positive breast cancer.

Thus, based on the relevance and prevalence of BC among patients in the Republic of Kazakhstan and the need to improve early cancer screening protocols, the research goal is to study single SNPs in patients with BC in the Kazakh population.

Currently, it is known that more than 10% of breast cancer cases are hereditary, with almost 5% of diagnosed cases associated with already identified high penetrance traits inherited through the autosomal dominant type (Wendt and Margolin, 2019). Recent advances in cancer genetics have led to increased awareness of personalized risk assessment in the primary prevention and screening of BC (Elezaby et al., 2019). Risk assessment is a multistep process that aims to identify patients at increased risk of developing breast cancer who are eligible to participate in intensive cancer screening protocols or may be offered preventive measures after referral for formal genetic testing.

The most prominent cause of hereditary BC is fetal prenatal mutations that occur in genes responsible for predisposition to the development of BC. This group of genes includes *BRCA1, BRCA2, TP53, CHEK2, PTEN, ATM*, and *PPM1D* (Mahdavi et al., 2019; Nagarajan et al., 2020). Genes associated with the possibility of developing BC can be divided into two groups: genes with high and low penetrance, each of which interacts with several other genes and environmental factors. The most common marker genes for the development of breast cancer are *BRCA1* and *BRCA2* (Mavaddat et al., 2019), which have fundamental functions in the repair of homologous DNA (deoxyribonucleic acid). Loss of heterozygosity, which is accompanied by inherited mutations in *BRCA1* or *BRCA2* (Dorling et al., 2021), increases chromosomal instability and the probability of developing a cancerous process. Most pathological mutations in the *BRCA1* and *BRCA2* genes are small deletions or insertions that cause the translation of a shortened protein (Angus et al., 2019; Mahdavi et al., 2019) . Protein 53 (*TP53*) regulates the cell cycle and is involved in DNA repair, apoptosis, cell aging, and metabolism. Female *TP53* mutation carriers have an almost 100% lifetime risk of developing cancer despite their ethnicity {Shahbandi, 2020). Protein 53 (*TP53*) regulates the cell cycle and is involved in DNA repair, apoptosis, cell aging, and metabolism. Female *TP53* mutation carriers have an almost 100% lifetime risk of developing cancer despite their ethnicity {Shahbandi, 2020; Elezaby et al., 2019). 

Racial and ethnic differences in breast cancer morbidity and mortality remain largely unknown, but possible risk factors include the socioeconomic status of the patient, the late stage of breast cancer at the time of diagnosis, biological and genetic differences in tumors, and different access to medical care. A 2022 genotypic study of the Kazakhstan western regions with diagnosed BC (Aitmagambetova, 2022) showed that luminal type A is the most common variant of BC in women in the Kazakh population (over 50% of cases). A retrospective analysis of breast cancer detection among women living in metropolitan cities of the Republic of Kazakhstan in 2009-2018 (Igissinov et al., 2019) showed a statistically significant trend in the increase in the detection of breast cancer but at the same time a decrease in the rates of mortality. Another study concerning the terms of detection and effective treatment of BC in Kazakhstan (Chukmaitov et al., 2018) shows that the patients treated in the regions of the country located further from Almaty had a higher risk of delayed treatment. However, the risk of an untimely diagnosis of breast cancer was higher in patients treated in Almaty. Despite the problems with organizing timely treatment for patients in the regions of the country, analysis of the effectiveness of screening programs (Kulkayeva et al., 2021) showed the effectiveness in the form of diagnosis of more than 35% of cases of breast cancer.

Several researches have been conducted in the Kazakh population. Li et al., 2007 examined the distribution of MDR1 and CYP3A5 SNPs within three distinct ethnic groups in mainland China: the Han, Uygur, and Kazakh populations. The results showed that the frequency of the MDR1 3435T variant was significantly higher in the Uygur population (52.8%) compared to the Kazakh (39.8%) and Han (37.9%) Chinese groups (p < 0.01, Fisher’s exact test). However, there was no significant difference in the frequencies of the MDR1 1236T and 2677T/A variants between the Han, Uygur, and Kazakh populations. But there are still few studies to outline this topic in Kazakh region. This study’s novelty lies in its specific focus on the Kazakh population and the examination of gene polymorphisms as potential breast cancer risk factors within this group. By shedding light on the genetic basis of breast cancer in the Kazakh population, this research aims to advance understanding of the disease and pave the way for tailored interventions and improved healthcare outcomes.

## Materials and Methods

The research is prospective, comparative, and diagnostic in nature. The first research step is the analysis of statistical and research databases to select polymorphisms associated with the development of BC in women. For this purpose, dbSNP, Snapshot, SNPedia, SNPdbe, and HapMap (National Library of Medicine, 2022) databases were used. Furthermore, two groups of patients were formed for the study of SNPs associated with breast cancer in the Kazakh population. dbSNP (Single Nucleotide Polymorphism Database) is a comprehensive database maintained by the National Center for Biotechnology Information (NCBI). It contains information about single nucleotide variations in the human genome. You can access dbSNP through the NCBI website. A database snapshot is a read-only, static view of a SQL Server database (the source database). The database snapshot is transactionally consistent with the source database as of the moment of the snapshot’s creation. A database snapshot always resides on the same server instance as its source database. SNPedia is a community-curated resource that catalogs information about SNPs and their associations with various traits and diseases. It can be accessed through the SNPedia website. SNPdbe—SNP database of effects, with predictions of computationally annotated functional impacts of SNPs. Database entries represent nsSNPs in dbSNP and 1000 Genomes collection, as well as variants from UniProt and PMD. SAASs come from >2600 organisms; ‘human’ being the most prevalent. HapMap is a database that provides information on human genetic variation, particularly focusing on haplotypes. Group No. 1 included 200 women in the Kazakh population with diagnosed and confirmed BC. Criteria for inclusion of patients in the Group No. 1 were age 18 years and more, morphologically confirmed adenocarcinoma of the breast with the immunohistochemical study of the expression of adenocarcinoma markers, general condition of the patient under ECOG (Eastern Cooperative Oncology Group) scale from 0 to 2 (Neeman et al., 2019), life expectancy more than 6 months, and absence of any psychological, family, sociological or geographical conditions, potentially preventing the research according to ECOG methodology. All patients signed a written informed consent to participate in the research. In the absence of a morphologically confirmed diagnosis, refusal to sign a written consent, or refusal to use the patient’s clinical data in the study – the patient did not take part in the research.

Group No. 2 included 200 somatically healthy women in the Kazakh population with no cancer pathology in their anamnesis at the time of participation in the research. The state database “Electronic Registry of Cancer Patients of the Republic of Kazakhstan” (Electronic Registry of Cancer Patients of the Republic of Kazakhstan, 2022) was used to confirm the anamnestic data on cancer diseases. DNA was isolated from peripheral blood by desalinization. Genotyping of the SNPs in patients’ DNA was performed using specially manufactured SNP platforms and reagents (total number – 128): TaqMan® OpenArray® genotyping plate, Custom format 128; 384-well OpenArray® sample plates; OpenArray® loading guides; TaqMan® OpenArray® Genotyping Master Mix. All steps were performed on “Real-time PCR system Quant Studio 12 R Flex” equipment. Statistical analysis of the obtained genetic study data was performed using the StatTech computer program, version 2.5.9 (software developer – “StatTech”, limited liability company). Quantitative data were analyzed for compliance with a normal ranking according to the Shapiro-Wilk criterion (when the number of patients tested was less than 50) or the Kolmogorov-Smirnov criterion (when the number of patients tested was more than 50).

If no normal ranking of indicators was available, quantitative data were presented using median (Me) and lower and upper quartiles (Q1-Q3). Categorical measures were presented with absolute values and percentages. Percentages were compared in four-field contingency table analyses using Pearson’s chi-square test (for expected phenomenon values greater than 10), and Fisher’s exact test (for expected phenomenon values less than 10). Percentages were compared in the analysis of monopole correlation tables using the nonparametric method - Pearson’s chi-square test. The level of significance or statistical significance was <5% (p<0.05) with a corresponding 95% confidence interval.

The research was conducted under ethical provisions in the sphere of biomedical research of the Republic of Kazakhstan, as well as under national and international guidelines and protocols on the ethics of medical research with human participants. Ethical principles of the Helsinki Declaration on conducting medical research with the participation of human subjects were observed. The research design and methodology were approved by the ethical commission of the Asfendiyarov Kazakh National Medical University (protocol No. 17 of 14.03.2017). Results may be limited in generalizability to other populations or nations outside of Kazakhstan.

## Results

*Analysis of polymorphisms associated with the development of breast cancer*

The first block of research results addresses the analysis of statistical and scientific databases to identify polymorphisms associated with the risk of developing and prognosis of breast cancer in women. Based on database processing, the following genotypic polymorphisms were selected, whose association with the development of BC was determined through evidence-based clinical studies of women from different populations. To comprehend the functional work of particular polymorphisms, they were categorized into biotin-coding proteins (Table 1), long noncoding RNAs (ribonucleic acids), and nonstop degradation (Table 2).

The selection results shown in the table demonstrate the extensive research of human genome variants for preventive detection of predisposition to breast cancer and can also be used for personalized patient management protocols.

*Genotyping results*

The results of the second stage of the research address the determination of polymorphisms with statistically significant differences in the group of breast cancer patients and the control group of conditionally healthy women in the Kazakh population using the process of genotyping of the obtained biomaterial. Genetic analyses revealed that single-nucleotide base substitutions may be most characteristic of women with breast cancer or never occur in this group of patients. The overall state of the gene polymorphism variants detected (Table 3) indicates a limited number of SNPs characteristic of the Kazakh population. 

The G/G rs55886062 homozygous polymorphism variant was identified only in the group of breast cancer patients in most cases (81.0%). The homozygous C/C type rs3918290 genotype was detected only in the group of BC patients, which may indicate a high risk of disease development in persons with this genotype. The single nucleotide variant rs12721655 with homozygous A/A genotype was revealed in 76.0% of cases in the group of Kazakhstan population of breast cancer patients. The rs4987117 polymorphism variant with the homozygous C/C genotype was detected in 74.0% of cases in BC patients. The heterozygous A/A variant of the rs2229774 polymorphism was detected only in the group of BC patients and was completely absent in the group of somatically healthy women. The G/G polymorphism rs11203289 and the rs34945627 variant with the C/C genotype were also not detected in conditionally healthy women. The presence of a heterozygous C/T genotype in rs2227945 may indicate a low risk of BC. The recessive variant of the C/C genotype of the rs4415084 polymorphism is found in 24.5% of breast cancer patients. The homozygous genotype of G/G variant rs137852576 as well as SNP rs11571833 with A/A genotype may be the predictor of breast tumor development in women of the Kazakh population. The C/C genotype of the rs80359062 polymorphism is not found in the No. 2 group, but it is detected in 66.5% of cases of breast cancer patients.

The T/T genotype with the recessive rs11571746 type polymorphism was not identified in presumably healthy women. The rs80357382 polymorphism with the heterozygous S/T genotype in 12.5% of cases was identified in a relatively healthy group of patients may indicate a low risk of breast cancer in the Kazakh population. A Heterozygous S/T genotype with rs9934948 polymorphism was not detected in the group of breast cancer patients. The presence of the G/G genotype with rs3218707 variation only in the group of conditionally healthy women may indicate a low risk of BC development. The A/A genotype with the rs17879961 polymorphism variant is associated with the development of BC in 75% of cases, as it has been detected exclusively in the group of BC patients. The G/G homozygous genotype for rs4778137 was detected only in the group of BC patients. In the rs1800058 polymorphism, the homozygous T/T genotype was not detected in conditionally healthy individuals.

The evaluation of rs4646 polymorphism depending on the research group revealed significant differences (p=0.028, hereinafter Pearson chi-square method was used). A prognostic model for determining the probability of detecting a tumor depending on SNP rs4646 was developed by binary logistic regression, the regression model was statistically significant (p=0.028). Based on the value of the Nigelkirk determination coefficient, the model explains 2.3% of the observed variance of the study group. When assessing the dependence of the probability of patients in group No. 2 on the value of the logistic function P using ROC (receiver operating characteristic) analysis, the area under the ROC curve was obtained, which was 0.567±0.028 with 95% CI (confidence intervals): 0.513-0.622. The resulting model was statistically significant (p=0.010). The threshold value of the logistic P function at the cut-off point, corresponding to the maximum value of the Juden index, was 0.598. The sensitivity and specificity of the model were 46.8% and 66.0%, respectively.

The research of the SNP indicator of type rs1065852 revealed statistically significant differences (p<0.001) (the resulting regression model is statistically significant, p<0.001). Based on the value of the Nigelkirk determination coefficient, the model explains 7.5% of the observed variance in group No. 1. When estimating the dependence of the probability in group No. 2 on the value of the logistic function P using ROC analysis, the area under the ROC curve was 0.605±0.028 with 95% CI: 0.551-0.659 with sensitivity and specificity of 93.1% and 23.0% in the groups, respectively.

A comparison of the rs4244285 type polymorphism index depending on the research group revealed statistically significant differences (p=0.002). The prognostic model for determining the probability of the research group depending on the rs4244285 index was statistically significant (p=0.001). Based on the value of the Nigelkirk determination coefficient, the model explains 4.3% of the observed variance. In a ROC analysis, a statistically significant (p<0.001) correlation under the ROC curve was obtained between the probability of detection in conditionally healthy women, which was 0.579±0.028 with a 95% CI: 0.525-0.634. The sensitivity and specificity of the model were 42.1% and 71.5%.

The rs67376798 analysis for heterozygous A/T genotype was 11.3 times higher in group No. 2 than in group No. 1. The differences in the odds of detecting SNP were statistically significant (95% CI: 5.253-24.158). Based on the value of the Nigelkirk determination coefficient, the model explains 18.1% of the observed variance of the study group. The heterozygous A/T genotype was accompanied by an increased probability in the No. 2 group when assessing the effect of rs67376798. The area under the ROC curve was 0.640±0.027 with a 95.0% CI: 0.587-0.692 (the resulting model was statistically significant, p<0.001). The threshold value of the logistic P function at the limiting point corresponding to the maximum value of the Juden index was 0.896. The sensitivity and specificity of the model were 31.9% and 96.0%, respectively.

The A/A genotype with the rs6504950 polymorphism shows statistically significant differences (p=0.042) depending on the study group. This type of SNP is characteristic only for BC patients, but when evaluating the dependence of its detection probability in group No. 2 on the value of the logistic P function using ROC analysis, the resulting model was not statistically significant (p=0.137). When evaluating the rs2229774 polymorphism index, statistically significant differences (p=0.001) were found depending on the study group. Genotypes A/G and A/A have been detected only in group No. 2. The area under the ROC curve was 0.530±0.028 at 95.0% CI: 0.475-0.585 with statistically significant differences (p<0.001). The sensitivity and specificity of the model were 100.0% and 6.0%, respectively.

When analyzing the SNP rs1800056 index, statistically significant differences (p<0.001) were found depending on the study group, which were based on the values of the Nigelkirk determination coefficient. The model explained 15.4% of the observed variance of the study group. The S/T and S/S genotype were accompanied by an increase in the probability of presence in the No. 2 group when assessing the effect of the SNP index “rs1800056”. The area under the ROC curve was 0.617±0.027 with a 95.0% CI: 0.564-0.671. The resulting model was statistically significant (p<0.001). The sensitivity and specificity of the model were 26.4% and 97.0%, respectively.

The analysis of the rs16942 index depending on the research group demonstrated significant differences (p=0.026), with the odds of detecting the heterozygous S/T genotype being 1.6 times higher in the conventionally healthy women compared to the group of breast cancer patients. The differences in odds were statistically significant (95.0% CI: 1.055-2.416). The regression model was statistically significant (p=0.026) and the area under the ROC curve was 0.551±0.028 with 95.0% CI: 0.496-0.606. The sensitivity and specificity of the model were 72.7% and 37.5%, respectively.

Significant differences (p=0.004) were found in the cases of rs4987047-type SNPs depending on the research group: the odds of heterozygous A/T genotype was 1.845 times lower in the No. 2 group compared to the SNPs group, differences in odds were statistically significant (95% CI: 0.355-0.826). The resulting prognostic regression model (p=0.004) of the rs4987047 relationship, was based on the value of the Nigelkirk determination coefficient. The model explained 2.6% of the observed variance of the research group. The ROC analysis was used to estimate the dependence of the probability of detection in conditionally healthy women of the No. 2 group on the value of the logistic P function: the area under the ROC curve was 0.565±0.028 with a 95% CI: 0.510-0.620. The sensitivity and specificity of the model were 75.5% and 37.5%, respectively.

A comparative analysis of the frequency of polymorphisms in the groups with favorable and unfavorable prognoses of breast cancer was performed. During the analysis of the data obtained depending on the ranking by age of the patients, no statistically significant differences by Pearson’s chi-square method were found, which confirms the given statement that the SNPs are relatively stable and cannot be caused by phenotypic changes alone.

To assess the influence of the hereditary factor on the development of breast cancer, single nucleotide base substitutions depending on the presence or absence of a hereditary history were analyzed. Comparison of rs2740574, rs13389423, and rs616488 depending on the burdened heredity revealed significant differences (p=0.020, p=0.012, p=0.020, respectively). The Heterozygous C/T genotype by the rs2740574 polymorphism was detected only in the group with heredity. The heterozygous A/G genotype was significantly more frequent in the group with a hereditary history of the rs13389423 polymorphism and was not found in patients without a hereditary history of the rs616488 polymorphism.

An indirect method of assessing the aggressiveness of breast cancer is an analysis of the substitution of single-nucleotide polymorphisms according to the extent of the disease at the time of diagnosis. The actual point of assessment may depend on the availability of diagnostic capabilities of the hospital, the place of residence, and the social status of the patient. Despite this, the analysis and statistical processing of the data obtained highlights several SNPs (Table 4), the genotypic detection of which in patients with or without a history of a poor family history places them in the high-risk group for developing BC in the focus of the Kazakh population and allows them to receive additional diagnostic methods and active preventive surveillance.

As a result of a comparison of SNPs of the rs144848 and rs1143684 types depending on the degree of prevalence, statistically significant differences (p=0.017 and p=0.029, respectively) were established. The heterozygous A/C genotype in the rs144848 polymorphism is less characteristic for locally advanced and advanced forms of BC, as well as for the C/C genotype with the rs1143684 polymorphism. lThe study involved an analysis of single nucleotide base substitutions depending on the histological pattern of breast adenocarcinoma and the degree of tumor malignancy. Depending on the histological grade of malignancy we failed to establish statistically significant using Pearson’s chi-square and Fisher’s exact criterion methods.

Analysis of base substitutions in polymorphisms depending on tumor molecular subtype (established immunohistochemical method) showed statistically significant differences of rs1143684 polymorphism depending on tumor molecular subtype (p=0.044). Homozygous genotype T/T rs1143684 was significantly more frequently encountered in luminal type B adenocarcinoma, while the C/C rs1143684 genotype was significantly more frequent in the group of patients with Her2neu-positive tumor type. No correlation with luminal type A or triple-negative type was found. The index of proliferative activity is a histological indicator of tumor aggressiveness and possible invasion. Comparison of SNP index rs3803662 and rs6678914 as a function of proliferative activity revealed statistically significant differences (p=0.021 and p=0.035 respectively by Pearson chi-square method). The G/G genotype by the rs3803662 polymorphism was significantly less common in tumors with high proliferative activity. In turn, the heterozygous A/G genotype of the rs6678914 polymorphism was most common in tumors with low proliferative activity.

**Table 1:** Protein-coding Polymorphisms Associated with the Development of Breast Cancer in Women

Type of single nucleotide polymorphism	Explanation	Biotype	Sequence	Possible action
rs7853758	SLC28A3	Protein encoding	synonymic variant	Usage in medical reactions
rs2740574	CYP3A4	Protein encoding	upstream gene variant	Usage in medical reactions, benign
rs1800566	NQO1	Protein encoding	missense-mutation	Usage in medical reactions, pathogenetic, risk factors, and additional values are studied
rs1045642	ABCB1	Protein encoding	synonymic variant	Usage in medical reactions, benign, additional values being studied
rs4646	CYP19A1	Protein encoding	a variant of the untranslated area 3 prime	Benign, usage in medical reactions
rs1065852	CYP2D6	Protein encoding	missense-mutation	Usage in medical reactions, benign, additional values being studied
rs55886062	DPYD	Protein encoding	missense-mutation	Usage in medical reactions, pathological
rs17863783	UGT1A6	Protein encoding	synonymic variant	Benign
rs4244285	CYP2C19	Protein encoding	synonymic variant	Usage in medical reactions, benign, additional values being studied
rs67376798	DPYD	Protein encoding	missense-mutation	Usage in medical reactions
rs3918290	DPYD	Protein encoding	Splicing variant	Usage in medical reactions, pathological
rs12721655	CYP2B6	Protein encoding	missense-mutation	Usage in medical reactions, benign, additional values being studied
rs2032582	ABCB1	Protein encoding	missense-mutation	Usage in medical reactions, benign, additional values being studied
rs2032582	ABCB1	Protein encoding	missense-mutation	Risk factors, usage in medical reactions, benign, additional values being studied
rs2032582	ABCB1	Protein encoding	missense-mutation	Risk factors, usage in medical reactions, benign, additional values being studied
rs2032582	ABCB1	Protein encoding	missense-mutation	Risk factors, usage in medical reactions, benign, additional values being studied
rs12762549	GRCh38	Protein encoding	intergenic variant	Risk factors, usage in medical reactions, benign, additional values being studied
rs4973768	SLC4A7	Protein encoding	the variant of the untranslated area 3 prime	Risk factors, usage in medical reactions, benign, additional values being studied
rs4987117	BRCA2	Protein encoding	missense-mutation	Mainly benign
rs2046210	GRCh38	Protein encoding	intergenic variant	Mainly pathological
rs6504950	STXBP4	Protein encoding	intron variant	Additional values are studied
rs438034	CENPF	Protein encoding	missense-mutation	Additional values are studied
rs111367604	BARD1	Protein encoding	missense-mutation	Additional values are studied
rs2229774	RARG	Protein encoding	missense-mutation	Additional values are studied
rs2227945	BRCA1	Protein encoding	missense-mutation	Benign
rs121434592	AKT1	Protein encoding	Missense-mutation, Splicing variant	Mainly pathological
rs139785364	BARD1	Protein encoding	missense-mutation	Additional values are studied
rs11203289	SDHB	Protein encoding	missense-mutation	Benign
rs12210538	SLC22A16	Protein encoding	missense-mutation	Mainly pathological
rs714368	SLC22A16	Protein encoding	missense-mutation	Mainly pathological
rs1800056	ATM	Protein encoding	missense-mutation	Benign
rs16942	BRCA1	Protein encoding	missense-mutation	Benign
rs1042522	TP53	Protein encoding	missense-mutation	Benign, usage in medical reactions
rs34945627	TNFRSF11A	Protein encoding	missense-mutation	Mainly pathological
rs8133052	CBR3	Protein encoding	missense-mutation	Mainly pathological
rs3803662	TOX3	Protein encoding	upstream gene variant	Mainly pathological
rs33927012	SDHB	Protein encoding	missense-mutation	Benign
rs1799966	BRCA1	Protein encoding	missense-mutation	Benign
rs10824792	MBL2	Protein encoding	a variant of the untranslated area 3 prime	Benign
rs11571747	BRCA2	Protein encoding	missense-mutation	Mainly benign
rs3817198	LSP1	Protein encoding	intron variant	Mainly benign
rs137852576	AR	Protein encoding	missense-mutation	Pathological
Type of single nucleotide polymorphism	Explanation	Biotype	Sequence	Possible action
rs11571833	BRCA2	Protein encoding	nonsense mutation	Mainly benign
rs80359062	BRCA2	Protein encoding	missense-mutation	Mainly pathological
rs3218536	XRCC2	Protein encoding	missense-mutation	Benign
rs80357382	BRCA1	Protein encoding	missense-mutation, Splicing variant	Pathological
rs11571746	BRCA2	Protein encoding	missense-mutation	Mainly benign
rs28934577	TP53	Protein encoding	missense-mutation	Mainly pathological
rs3798577	ESR1	Protein encoding	a variant of the untranslated area 3 prime	Mainly benign
rs12248560	CYP2C19	Protein encoding	missense-mutation	Risk factors, pathological
rs2981582	FGFR2	Protein encoding	intron variant	Mainly benign
rs1219648	FGFR2	Protein encoding	intron variant	Mainly benign
rs2981578	FGFR2	Protein encoding	intron variant	Mainly benign
rs6678914	LGR6	Protein encoding	intron variant	Risk factors, usage in medical reactions, benign
rs2290203	PRC1	Protein encoding	intron variant	Risk factor
rs4987047	BRCA2	Protein encoding	missense-mutation	Mainly benign
rs6001930	MRTFA	Protein encoding	intron variant	Risk factors, usage in medical reactions, benign
rs13389423	BARD1	Protein encoding	missense-mutation	Value is studied
rs11045585	SLCO1B3	Protein encoding	intron variant	Risk factors, usage in medical reactions, benign
rs351855	FGFR4	Protein encoding	missense-mutation	Mainly pathological
rs13387042	LOC105373874	Protein encoding	intergenic variant	Risk factors, usage in medical reactions, benign
rs1799983	NOS3	Protein encoding	missense-mutation	Mainly pathological, the risk factor
rs3218707	ATM	Protein encoding	missense-mutation	Benign
rs3218695	ATM	Protein encoding	missense-mutation	Benign
rs121912658	TP53	Protein encoding	missense-mutation	Mainly pathological
rs137852985	BRIP1	Protein encoding	missense-mutation	Mainly pathological
rs17530068	LOC105377871	Protein encoding	intergenic variant	Risk factors, usage in medical reactions, benign
rs11540652	TP53	Protein encoding	missense-mutation	Mainly pathological
rs121917739	RAD51	Protein encoding	missense-mutation	Mainly pathological
rs17879961	CHEK2	Protein encoding	missense-mutation	Risk factors, conflicting interpretations of pathogenicity, pathological
rs4778137	OCA2	Protein encoding	intron variant	Benign
rs4986761	ATM	Protein encoding	missense-mutation	Benign
rs1800057	ATM	Protein encoding	missense-mutation	Benign
rs3092856	ATM	Protein encoding	missense-mutation	Benign
rs1800058	ATM	Protein encoding	missense-mutation	Benign, value is studied, and conflicting interpretations of pathogenicity
rs1799950	BRCA1	Protein encoding	missense-mutation	Benign, the value is studied
rs1801426	BRCA2	Protein encoding	missense-mutation	Benign, the value is studied
rs1799954	BRCA2	Protein encoding	missense-mutation	Benign
rs144848	BRCA2	Protein encoding	missense-mutation	Benign, the value is studied
rs766173	BRCA2	Protein encoding	missense-mutation	Benign
rs1143684	NQO2	Protein encoding	missense-mutation	Overall survival and progression-free survival
rs1045485	CASP8	Protein encoding	missense-mutation	Protective, benign
rs616488	PEX14	Protein encoding	intron variant	Risk factors, usage in medical reactions, benign
rs1054135	FABP4	Protein encoding	the variant of the untranslated area 3 prime	Risk factors, usage in medical reactions, benign
rs1787991	FECH	Protein encoding	intron variant	Risk factors, usage in medical reactions, benign
rs121908984	SDHD	Protein encoding	missense-mutation	Value is studied
rs1800470	TGFB1	Protein encoding	missense-mutation	Risk factor, benign
rs199476086	BMPR1A	Protein encoding	missense-mutation	Pathological
rs2227924	ATM	Protein encoding	missense-mutation	Pathological
rs2228455	BARD1	Protein encoding	missense-mutation	Risk factor
rs28934578	TP53	Protein encoding	missense-mutation	Pathological
rs28934874	TP53	Protein encoding	missense-mutation	Pathological
rs3135718	FGFR2	Protein encoding	intron variant	Usage in medical reactions
rs3892097	CYP2D6	Protein encoding	Splicing variant	Benign, usage in medical reactions
rs3918242	MMP9	Protein encoding	upstream gene variant	Risk factor
rs80357540	BRCA1	Protein encoding	intron variant	Pathological
rs4986850	BRCA1	Protein encoding	missense-mutation	Benign
rs62625308	BRCA1	Protein encoding	missense-mutation	Value is studied
rs757229	GPX4	Protein encoding	upstream gene variant	Risk factor
rs7895676	FGFR2	Protein encoding	intron variant	Risk factor
rs80357914	BRCA1	Protein encoding	translational frameshift	Pathological
rs80357064	BRCA1	Protein encoding	missense-mutation	Pathological
rs80357540	BRCA1	Protein encoding	intron variant	Pathological
rs80357629	BRCA1	Protein encoding	translational frameshift	Risk factor
rs80357780	BRCA1	Protein encoding	intron variant	Pathological
rs80357906	BRCA1	Protein encoding	translational frameshift	Pathological, risk factor
rs80359550	BRCA2	Protein encoding	translational frameshift	Pathological, risk factor

**Table 2:** Additional Types of Polymorphisms Associated with the Development of Breast Cancer in Women

Type of single nucleotide polymorphism	Explanation	Biotype	Sequence	Medical meaning
rs16902094	CASC8	lncRNA	intron variant	Risk factor, benign
rs620861	CASC8	lncRNA	intron variant	uncertain significance
rs13281615	CASC8	lncRNA	intron variant	uncertain significance
rs4415084	LINC02224	lncRNA	upstream gene variant	Mainly pathological
rs3784099	RAD51B	lncRNA	intron variant	Mainly benign
rs9934948	LINC01568	lncRNA	downstream gene variant	Benign
rs12922061	CASC16	lncRNA	intron variant	Risk factor. benign
rs11249433	EMBP1	transcribed raw pseudogene	intron variant	Risk factor. benign
rs889312	GRCh38	promoter	regulatory variant	Risk factor. benign
rs713041	GPX4	Non-stop degradation	missense-mutation	Mainly pathological

**Table 3:** Identified Types of Single-nucleotide Polymorphisms in Patients of the Research Groups

SNP type	Group No. 1 (%)	Group No. 2 (%)
rs4646	88.70	not detected
rs55886062	81.00	not detected
rs3918290	62.30	not detected
rs12721655	76.00	not detected
rs4987117	74.00	not detected
rs2229774	52.40	not detected
rs11203289	40.00	not detected
rs34945627	32.80	not detected
rs2227945	17.00	not detected
rs4415084	24.50	not detected
rs137852576	87.60	not detected
rs11571833	84.90	not detected
rs80359062	66.50	not detected
rs11571746	39.20	not detected
rs80357382	30.00	12.50
rs3218707	not detected	16.00
rs17879961	75.00	not detected
rs4778137	69.50	not detected
rs1800058	48.00	not detected
rs1065852	92.00	not detected
rs4244285	86.00	5. 0
rs67376798	88.00	not detected
rs6504950	87.00	6.00
rs1800056	95.60	12.50
rs16942	63.00	2.60
rs2740574	23.00	not detected
rs13389423	26.00	not detected

**Table 4:** Single-nucleotide Polymorphisms Denoting Sensitivity and Specificity in Predicting Breast Tumor Risk

Polymorphism type	p depending on the group	The region under the ROC curve	p depends on the value of the logistic function	Sensitivity (%)	Specificity (%)
rs4646	p=0.028	0.567±0.028 with 95% CI: 0.513-0.622	p=0.010	46.80	66.00
rs1065852	p<0.001	0.605±0.028 with 95% CI: 0.551-0.659	p<0.001	93.10	23.00
rs4244285	p=0.002	0.579±0.028 with 95% CI: 0.525-0.634	p<0.001	42.10	71.50
rs67376798	p<0.001	0.640±0.027 with 95% CI: 0.587-0.692	p<0.001	31.90	96.00
rs6504950	p=0.042	0.630±0.027 with 95% CI: 0.587-0.634	p=0.137	77.80	28.00
rs2229774	p=0.001	0.530±0.028 with 95% CI: 0.475-0.585	p<0.001	100.00	6.00
rs1800056	p<0.001	0.617±0.027 with 95% CI: 0.564-0.671	p<0.001	26.40	97.00
rs16942	p=0.026	0.551±0.028 with 95% CI: 0.496-0.606	p=0.027	72.70	37.50
rs4987047	p=0.004	0.565±0.028 with 95% CI: 0.510-0.620	p=0.004	75.50	37.50

## Discussion

The analysis of SNP variants associated with the development of BC in women in the Kazakh population shows a wide range of possible genotype variants in patients, regardless of family history. In most cases, polymorphisms associated with protein-coding or other functions carry the potential threat of comorbid conditions and specific drug reactions, which may complicate the application of treatment protocols. Thus, by screening for cancer or identifying patients at risk of developing BC using genotypic testing, supervising physicians can broaden the field of vision of potential patient diseases and improve treatment outcomes.

A total of 117 SNP variants that are pathogenetically associated with the possible development of BC or are potential risk factors were selected. As a result of genetic studies in both groups (a control group with relatively healthy patients without a history of cancer and a group of patients with already diagnosed adenocarcinoma-type BC), 27 polymorphisms were found in the cohort of studied patients of the Kazakh population. The limited number of identified variants indicates a connection between the ethnic peculiarities of patients with the possibility to develop BC at a particular age typical for a given population, which is emphasized by modern scientific works (Ginsburg et al., 2020; Louro et al., 2019). SNPs with statistically significant differences for both groups of patients in the Kazakh population are necessary for the research.

A homozygous variant of the G/G polymorphism rs55886062 was found in a group of patients diagnosed with breast cancer, which is consistent with recent studies in other populations. For example, studies of the effect of several polymorphisms, including rs55886062, on the efficacy of traditional pharmacotherapy of breast cancer in women show a relation of dehydrogenase gene polymorphisms (rs3918290, rs55886062, rs67376798, rs75017182) with an increased risk of toxicity and adverse effects of fluoropyrimidine use, including hepato- and nephrotoxicity (Cura et al., 2021). Also, the HLA-DQA1-02:01 allele shows a direct association with the development of hepatotoxicity in patients with breast cancer receiving lapatinib over the treatment protocol. Thus, the G/G rs55886062 SNP detected in the Kazakh population in patients with diagnosed breast cancer may improve the tolerability of pharmacotherapy through individual adjustment of the treatment protocol due to the detected polymorphism (as recommended by the latest scientific protocols of patient management (Hertz and Sahai, 2020), and for a relatively healthy group of women - be a direct risk factor of possible breast cancer development.

The homozygous S/T type rs16942 genotype was characteristic of patients with BC in the Kazakh population, which was also found in studies conducted in the Tunisian population (Hamdi et al., 2018). Researchers compared the frequency of identified Tunisian alleles and distribution patterns with other populations and performed a comprehensive evaluation of the functional effects of the SNP variants. It was shown that polymorphisms at loci 2p24, 4q21, 6q25, 9q31, 10q26, 11p15, 11q13, and 14q32 are quite frequent in the Tunisian population, and the frequency of rs13329835 and rs16942 is statistically significantly different between Tunisian and other populations. It was the S/T genotype with the rs16942 variant that was also detected in the Kazakh population, which indicates the importance of narrowing the search for possible UTIs when conducting screening programs in the population.

The A/G and A/A rs6504950 genotype was detected exclusively in women diagnosed with breast cancer and showed high detection sensitivity (over 75%). This SNP was also detected in a genotypic study of Taiwanese women: the alternating number of variations in the 17q23 rs6504950 locus appeared to be directly statistically associated with the progressive course of breast cancer in the population (Lin et al., 2020). A study of cancer samples showed a high number of variations and copies in susceptible loci 2q35, 3p24, 17q23, and FGFR2 in patients undergoing treatment. Women with breast adenocarcinoma had a relatively high number of 17q23 rs6504950 SNPs, which is consistent with the identified indices of this polymorphism in the Kazakh population. The multivariate analysis of this study showed that SNP 17q23 rs6504950 for the Taiwanese population was a risk factor for patients with breast cancer.

The results of genotyping of patients in the Kazakh population had a statistically significant relationship with the histological picture of the tumor examined using standard morphological and complementary immunohistochemical methods. The T/T rs1143684 variant was more frequently encountered in cases of diagnosed adenocarcinoma of luminal type B, i.e., hormone positive; the C/C rs1143684 genotype was typical for patients with Her2neu-positive tumors, which implies the use of targeted drugs for Her2neu receptors. The dependence of histological and phenotypic breast tumor profiles on patient genotype has been investigated in different populations and continues to be studied (Mahdavi et al., 2019; Megías-Vericat et al., 2021; Sirisena et al., 2018; Wendt and Margolin, 2019). It was investigated (Alimardani et al., 2021) that the G allele rs799917 and the G allele rs1713611 were statistically significantly associated with the age of patients (range 50-59 years) diagnosed first with breast cancer in the Sri Lankan population. The SNP rs13689 is associated with an estrogen-positive variant of breast cancer, and the SNP rs1130214 and rs2071002 have been associated with Her2neu-positive breast cancer in women. The C *BRCA2* rs15869 allele and the C CCND1 rs7177 allele were statistically likely to be associated with a high histological degree of tumor proliferation, eating high atypia, and invasion rates. In the study conducted in the Kazakh population, the G/G genotype by rs3803662 polymorphism was statistically less common in tumors with high proliferative activity (immunohistochemical marker Ki-67). In contrast to these data, some studies found no statistically significant differences between histopathological characteristics and molecular subtypes of breast cancer in the groups of patients with different histological types of breast adenocarcinomas and genotypically detected polymorphisms (Ahearn et al., 2019; Nuruev et al., 2023; Semianiv et al., 2021).

Research of a group of women in the Kazakh population diagnosed with breast cancer showed that genotype A/A rs17879961 was associated in more than 70.0% of clinical cases with the development of an invasive variant of breast cancer, as it was identified exclusively in the No. 1 patient group studied. The association of this SNP with an increased risk of developing breast cancer was also studied in a large-scale study on 280000 Asian and European female patients in 2019 and had a different trend in prevalence (Khoshravan et al., 2022; Yang et al., 2019). Of the most common SNP variants rs6435074 and rs6723097 in CASP8 4, rs17879961 in *CHEK2* 2, and rs2853669 in TERT 5 (telomerase reverse transcriptase), it was rs17879961 that was very rarely found in Asian patients. The other three variants showed statistically stable associations for both Asians and Europeans. The two intronic variants of the CASP8 SNPs rs6435074 and rs6723097 showed similar associations in European patients, but the SNP variant rs6435074 showed a stronger occurrence in Asians. There was also an association with the risk of developing breast cancer for the intronic SNP variant rs676387 in the HSD17B gene; another SNP variant rs4793090, which is in LD (linkage disequilibrium) with rs676387 in both Asians and Europeans, was associated with a high risk of breast cancer at the genome-wide level. 

In a study of a Kazakh population, the heterozygous A/C genotype in rs144848 was less characteristic of locally advanced and common forms of BC, and the heterozygous C/T genotype rs2740574 was found exclusively in the group with a history of heavy family history. Similar data were obtained in Brazilian populations (Dobbin et al., 2021). Three *BRCA2* gene variants: rs11571769, rs144848, and rs11571707 had a high frequency of occurrence, different from the frequency observed in the other populations evaluated. The rs11571707, rs11571769, and rs144848 of the *BRCA2* gene were statistically likely to be associated with the development of hereditary BC in the Brazilian and Latino American populations.

de Bruin et al., 2012 explored the experiences and challenges encountered when incorporating these genetic variants into existing risk models and clinical practice. As more SNPs linked to breast cancer risk are discovered and risk estimates are refined, the ultimate goal is to leverage this information to inform personalized decision-making in risk management. Breast cancer is a complex disease, with genetic alterations playing a pivotal role in its development. While rare, high-penetrance mutations, such as *BRCA1* and *BRCA2*, account for a minority of breast cancer cases, recent advancements in genomic sequencing technologies have opened new avenues for uncovering additional genetic modifiers that influence breast cancer risk. Among these, an increasing number of risk-associated single nucleotide polymorphisms (SNPs) are being identified. Although individually these SNPs often confer only a modest increase in risk, their cumulative effect, when acting in concert, can substantially alter an individual’s susceptibility to breast cancer (Mamedalieva et al., 2021; Rubins et al., 1992).

Studies Chahil et al., 2015 have yielded valuable insights into the genetic basis of common diseases, identifying numerous single nucleotide polymorphisms (SNPs) associated with disease risk. This study’s primary objective was to replicate previously published SNPs that exhibited statistical significance in the context of breast cancer risk within the Malaysian population. In this case–control study, were recruited a cohort of 80 subjects for each group from various healthcare institutions in Malaysia. A total of 768 SNPs were genotyped and meticulously analyzed to distinguish between risk-associated and protective alleles. Notably, were identified three SNPs that were associated with an elevated risk of breast cancer, while six SNPs exhibited a protective effect. Importantly, all nine SNPs demonstrated statistical significance (p ≤ 0.01), with five of them successfully replicating findings from prior studies.

Breast cancer, a multifaceted disease with diverse risk factors, remains a significant global health concern. Understanding these factors is pivotal for prevention, early detection, and personalized healthcare. Age, an immutable factor, plays a pivotal role in breast cancer risk. As we age, the likelihood of developing breast cancer increases significantly. Gender, another non-modifiable element, places women at higher risk, though men too can be affected. Genetics, a non-modifiable determinant, contributes profoundly. Family history, especially in first-degree relatives, and specific genetic mutations, such as *BRCA1* and *BRCA2*, elevate risk levels. Hormone-related factors are partly modifiable. Hormone replacement therapy, depending on type, duration, and timing, may elevate risk. Conversely, reproductive history, modifiable through family planning, influences risk; early menstruation, late menopause, and nulliparity or late childbirth increase susceptibility (Houshyari and Taghizadeh-Hesary, 2023; Svyatova et al., 2019). Breastfeeding, partly modifiable, contributes positively to risk reduction. Longer duration yields greater protection. Environmental and occupational exposures, partly modifiable, may heighten risk. Minimizing exposure, when possible, is a preventative step. In sum, breast cancer risk is a product of numerous interacting factors (Abil’dinova et al., 2003). Personalized risk assessment, early detection, and prevention strategies must consider this intricate interplay. Regular screenings and consultation with healthcare professionals ensure proactive management. By acknowledging the complexity of these risk factors, we can enhance our understanding and combat breast cancer more effectively.

Breast cancer, a formidable adversary, faces transformative change with cutting-edge diagnostics and treatments. Innovative diagnostics, including advanced imaging and genetic testing, enable early detection, offering a vital edge for timely, targeted interventions. Personalized therapies, minimizing side effects, redefine treatment effectiveness. Minimally invasive surgeries, refined adjuvant therapies, and image-guided treatments enhance care (Schapovalova et al., 2022; Zyuzkov et al., 2022). Clinical trials expand options, while patient-centered approaches prioritize holistic well-being. Screening improvements and patient empowerment fortify prevention. These innovations illuminate a path toward improved survival and enhanced quality of life in the breast cancer journey.

In conclusions, breast cancer is the most common type of cancer affecting women, covering a quarter of all cancers diagnosed. The analysis of databases of polymorphisms associated with the development of breast cancer in women and its comparison with the identified polymorphisms in the No. 1 and No. 2 groups showed an extensive list of single-nucleotide polymorphisms that are characteristic of the Kazakh population. The rs13389423 and rs2740574 type polymorphisms are statistically significantly associated with a high risk of breast cancer in patients with a history of poor family history. The rs1143684 polymorphism has a statistically significant association with the histological variant of adenocarcinoma of Her2neu positive or luminal type B, while the rs3803662 variant is associated with the development of tumors with high proliferative activity, that is, with an increased probability of metastases.

The panel of single nucleotide polymorphisms, consisting of the set rs2740574, rs13389423, rs616488, rs1143684, rs3803662, rs6678914, allows one to determine the group of unfavorable prognoses of breast cancer patients by hereditary, molecular genetic profile and tumor proliferative activity degree in the Kazakh population. The polymorphisms rs55886062, rs3918290, rs12721655, rs4987117, rs2229774, rs11203289, rs137852576, rs11571833, rs80359062 and rs11571746 occurred with a frequency of 40.0% and higher in the Kazakh population of breast cancer patients and may therefore be used as early risk group detection. The variants of detected single nucleotide polymorphisms rs4646, rs1065852, rs4244285, rs67376798, rs6504950, rs2229774, rs1800056, rs16942, rs4987047 in group No. 1 are statistically significant compared to control group No. 2 patients. Gene panel testing is necessary for identifying women with a high risk of breast cancer. Improvements in early detection and assessment of patients at high risk for breast cancer may play an important role in improving the individual prognosis of familial breast cancer risk and reducing mortality in the Kazakh population. Further study of genetic polymorphisms in women in the Kazakh population with diagnosed breast cancer should be directed toward studying the prevalence of certain types of polymorphisms in each region and their correlation with the age range of primary tumor diagnosis. The identified genetic polymorphisms can be used to assess individual breast cancer risk, guide tailored screening strategies, and inform personalized prevention measures for women in the Kazakh population.

## Author Contribution Statement

All authors contributed equally to the concept, literature search, writing manuscript, critical revision, and finalising the manuscript.
